# Correlation of retinal layer changes with vision gain in diabetic macular edema during conbercept treatment

**DOI:** 10.1186/s12886-019-1131-0

**Published:** 2019-05-31

**Authors:** Yupeng Xu, Yuan Qu, Yan Suo, Jian Gao, Xia Chen, Kun Liu, Xun Xu

**Affiliations:** 1 Department of Ophthalmology, Shanghai Key Laboratory of Ocular Fundus Diseases, Shanghai General Hospital, Shanghai Engineering Center for Visual Science and Photo Medicine, Shanghai Jiao Tong University School of Medicine, Shanghai, China; 2 Department of Ophthalmology, The First Affiliated Hospital of Anhui Medical University, Hefei, Anhui China; 3 Department of Ophthalmology, Shanghai Eye Diseases Prevention and Treatment Center/ Shanghai Eye Hospital , Shanghai, 200080 China

**Keywords:** Diabetic macular edema, Retinal layer thickness, Layer segmentation, Anti-VEGF, Conbercept

## Abstract

**Backgrounds:**

To assess the changes in individual retinal layer thickness and visual function associated with gains in visual acuity after an intravitreal conbercept injection in the diabetic macular edema (DME) on spectral domain optical coherence tomography (SD-OCT) and microperimetry during 1-year follow-up.

**Methods:**

Retrospective observational study. Twenty patients with clinically significant DME in the study eye were imaged by SD-OCT every 3 months and MP1 microperimeter in the third month while receiving anti-vascular endothelial growth factor (VEGF) (conbercept) treatment. In each patient, seven retinal layers were segmented in 98 scans covering a 6 mm × 6 mm area of the macula at baseline and during 1 year of treatment. An automatic, full-threshold microperimetry of the central field (10° × 10°, 40 stimulated points) with the MP1 microperimeter. Thickness and microperimetry changes were quantitatively measured and evaluated for their correlation with increases in visual acuity.

**Results:**

Although thicknesses of the inner nuclear layer (INL) and the outer nuclear layer (ONL) were reduced the most after treatment (*p* < 0.05), decreases of the ganglion cell layer (GCL) (*r* = 0.591, *p* = 0.006) and inner plexiform layer (IPL) (*r* = 0.663, *p* = 0.001) in central subfield area was associated with best-corrected visual acuity (BCVA) gain, and had the best estimation of BCVA gain (adjust *R*^*2*^ = 0.544). Mean macular sensitivity in the central subfield was also well correlated with BCVA gain (*r* = 0.531, *p* = 0.016).

**Conclusions:**

Neural recovery occurred after the resolution of edema during conbercept treatment, due to the decreases in GCL and IPL associating with gains in vision and improved microperimetry.

**Electronic supplementary material:**

The online version of this article (10.1186/s12886-019-1131-0) contains supplementary material, which is available to authorized users.

## Background

Diabetic retinopathy (DR) is one of the leading causes of vision loss among working-aged adults globally [1]. Of the estimated 382 million people with diabetes mellitus worldwide [2], approximately 35% have signs of DR; of these, a further one third of DR cases are vision-threatening DR, most of which are diabetic macular edema (DME) [3]. Prolonged hyperglycemia in DME patients causes hypoxia and inflammation, resulting in an upregulation of growth factors and cytokines, e.g. vascular endothelial growth factor (VEGF) [4] which is essential for causing vascular leakage and acts as an proinflammatory cytokine in the pathogenesis of DME [2]; thus, the intravitreal anti-VEGF injection is now a standard treatment for DME [5, 6].

The evaluation of retinal microstructures by spectral domain optical coherence tomography (SD-OCT) allows quantitative analysis of DME [7]. Total retinal thickness has often been used to monitor treatment effectiveness of anti-VEGF therapy for DME [8, 9]. Changes in the individual retinal layer might serve as a biomarker for response to treatment [10, 11]. A recent study compared the changes in layer thickness of DME patient after 1-year of anti-VEGF or cortisone and found that the decrease in retinal nerve fiber layer (RNFL) may have a possible impact on best-corrected visual acuity (BCVA) gain [12].

Conbercept (KH902; Chengdu Kanghong Biotech Co., Ltd., Sichuan, China) is a recombinant fusion protein that was designed as a receptor decoy and has high affinity for all VEGF isoforms and placental growth factor (PlGF) [13]; it is widely used in anti-VEGF therapy in China [14, 15]. To the best of our knowledge, no study has examined the effect of conbercept on individual layer changes in DME treatment.

Also, limited previous studies exam the function of retinal ganglion cells with combination to the BCVA changes and layer thickness changes. As signs of neurodegeneration are also prevalent in patients with diabetes, the impact of intravitreal medication on functional changes in macular needs to be investigated in detail. Microperimetry was widely used in the previous studies to exam the function of retinal ganglial cell (RGC) in macular and thus could be used as a biomarker of RGC function [16, 17].

This study aimed to measure the thickness from all retinal layers during 1-year follow-up to find correlations between visual acuity recovery and retinal layer thickness changes purely from the treatment effect. Microperimetry examination was also performed to further exam the function of RGC changes with BCVA gain. Our study might provide a new view on what might happen after 1-year treatment of anti-VEGF therapy with conbercept.

## Materials and methods

### Study design

This study is a retrospective single-center observational study. This study followed the tenets of the Declaration of Helsinki and was approved by the Ethics Committee of Shanghai General Hospital (No. 2016KY003). Data were obtained from the database of ophthalmology in Shanghai General Hospital. Written informed consent was obtained from all subjects.

### Participants

Patients with untreated clinically significant DME in the study eye without previous anti-VEGF drug or steroid treatment and without clinically significant macular edema in fellow eye were included in the study. A central subfield thickness of 320 μm for males and 305 μm for females was followed for clinically significant macular edema (for Heidelberg device) [18]. Intravitreal conbercept (0.5 mg/0.05 mL) injections were performed by one retinal specialist. Patients were followed-up for 12 months between August 2010 and December 2013. All patients received three injections in the first 3 months, and then a pro re nata (PRN) strategy was then followed. Each patient underwent a thorough ophthalmic examination, including BCVA, slit lamp observation, and SD-OCT measurements, before the first injection and at each follow-up visit. The exclusion criteria were the following: (1) any non–diabetes-related macular edema; (2) any ophthalmological or neurological disease that could affect or could have affected the visual acuity (uncontrolled glaucoma, uveitis, retinal macular traction or macular epiretinal membrane); and (3) an eye with a history that could potentially affect the retinal layer thickness, e.g., vitrectomy, laser photocoagulation and cataract extraction.

The BCVA was tested at baseline and monthly thereafter during 1-year follow-up to using Early Treatment Diabetic Retinopathy Study (ETDRS) charts at 4 m. The serum glycated hemoglobin level was assessed at baseline.

### Optical coherence tomography

A 5.4 × 5.4-mm area of the macular region centered on the fovea was examined by the Spectralis SD-OCT (Heidelberg Engineering, Heidelberg, Germany) with display mode; volume scans of 97 sections were centered on the fovea, and 5 B-scan images at each section were averaged. For standardization, all examinations were performed by one well-trained technician who was masked to the identity of the subjects and was not involved in the data analysis. A build-in automatic recognition system enabled scanning of precisely the same location during the follow-up examinations.

To identify eyes/patients with increased thickness in the central subfield, the protocol proposed by DRCR.net was followed. Foveal thickness was manually segmented by one retinal specialist (Y.S.) using the SD-OCT software (Spectralis version 6.3.2.0, Heidelberg Engineering, Heidelberg, Germany). All scans of a particular eye were graded consistently by the same reader to counteract a potential annotation bias. The whole retina was divided into three circles as follows: central subfield (diameter of 11,000 μm) and inner (diameter of 3000 μm) and outer (diameter of 6000 μm) rings. The inner and outer rings were divided into the superior, inferior, nasal and temporal regions, and the average retinal thickness in these total 9 regions was measured automatically. The Heidelberg Software recognizes retinal tissue interfaces and, using these landmarks, allows the software to handle the following retinal layers: retinal nerve fiber layer (RNFL), ganglion cell layer (GCL), inner plexiform layer (IPL), inner nuclear layer (INL), outer plexiform layer (OPL), outer nuclear layer (ONL), and the photoreceptor–retinal pigment epithelium complex (PR). All layers were first segmented using the built-in automated software, and manual correction of artifacts was then performed to obtain precise results.

### Microperimetry examination

Microperimetry was performed on all subjects using the MP1 Microperimeter. (version 1.7.0., Nidek Technologies, Gamagori, Japan) in the first visit and the 3-month follow-up after injection. To avoid bias resulting from instrument variability, all examinations were calibrated accurately by the same experienced technician. The following parameters were used: a fixation target consisting of a red ring 2° in diameter; white, monochromatic background at 4asb, stimulus size Goldman III with 200 ms projection time; Stimulus light attenuation was set at 10 dB, with a threshold 4–2-1 strategy [16, 17].

A macular 10° program is applied, in which 40 stimuli was used: the central 2° area (diameter of 600 μm) covered with 8 stimuli; superior, inferior, nasal and temporal areas were each targeted with 4 stimuli (totally 32 stimuli) located 6° (diameter of 1800 μm) and 10° (diameter of 3000 μm) from the center of the fovea. Mean sensitivity were calculated in central area (diameter = 1000 μm) and the four quadrants in the inner ring (diameter = 3000 μm).

### Statistical analyses

All statistical analyses were performed using SPSS version 18.0 for Windows software (SPSS, Chicago, IL, USA). All continuous results are expressed as the mean ± standard deviation. Correlations between the OCT parameters changes and injection times were evaluated using Spearman correlation coefficients because the injection times were not normally distributed while all other parameters were evaluated using Pearson correlation coefficients. We consider *r* > 0.5 as well positively correlated and *r* = 0.3 to 0.5 as moderate positively correlated. Univariate linear regression and stepwise backward multivariate linear regression analyses were performed. Individual clinical factors were subjected to univariate linear analysis and were subsequently entered in the multivariate analysis in a backward stepwise manner, if *p* < 0.1. The criterion for retention in the multivariate model was *p* < 0.05.

## Results

### Demographic and clinical characteristics

Twenty patients with DME, including 10 men and 10 women, were evaluated and followed up until 1 year. The baseline characteristic and follow-up results were revealed in Table 1. The mean age of patients with DME was 60.55 ± 8.65 years old. The serum glycated hemoglobin level was 7.13 ± 0.99%. Intraocular pressure was 15.79 ± 2.95 mmHg. Seven patients had hypertension. All patients were treated with conbercept monotherapy. The mean baseline visual acuity (EDTRS) of the treated eye was 57.50 ± 12.50 letters and increased by an average of 8.65 letters to 66.15 ± 14.47 letters at month 12 (*p* < 0.001). The average total retina thickness of the whole EDTRS grid decreased from 387.47 ± 88.08 μm at baseline to 347.44 ± 60.78 μm at month 12 (*p* = 0.003). The mean macular sensitivity increased from 8.17 ± 3.66db to 9.50 ± 4.18db after 3 months (*p* = 0.008). On average, patients received 6.60 intravitreal injections of conbercept during the one-year treatment.

**Table 1:** Overview of baseline demographic and clinical characteristics in our study

Visit	Age	Sex (Female)%	Injection times	Hypertension (%)	Glycated hemoglobin level (%)	BCVA (ETDRS)	CRT (μm)	MS (db)
Baseline	60.55 ± 8.65	50.00	6.60 ± 3.02	35.00	7.13 ± 0.99	57.50 ± 12.50	387.47 ± 88.08	8.17 ± 3.66
Follow-up	/	/	/	/	/	66.15 ± 14.47(Month12)	347.44 ± 60.78(Month12)	9.50 ± 4.18(Month3)

*BCVA* Best corrected visual acuity, *CRT* Central retinal thickness, *EDTRS* Early treatment diabetic retinopathy study, *IOL* Intraocular lens, *IOP* Intraocular pressure, *MS* Mean sensitivity of micrometry covering a 10° × 10° area of macula

### Changes in different layers’ thicknesses in 1 year of conbercept treatment

The center total retina thickness decreased from 482.05 ± 156.98 μm at baseline to 336 ± 92.84 μm at month 12 by an average of 146.05 μm (*p* = 0.002). The thickness changed more significantly within the inner ring than in the outer ring of the EDTRS grid (*p* < 0.05). The average thickness of inner ring decrease by an average of 75.20 μm and outer ring decreased by an average of 33.58 μm.

**Fig. 1:**
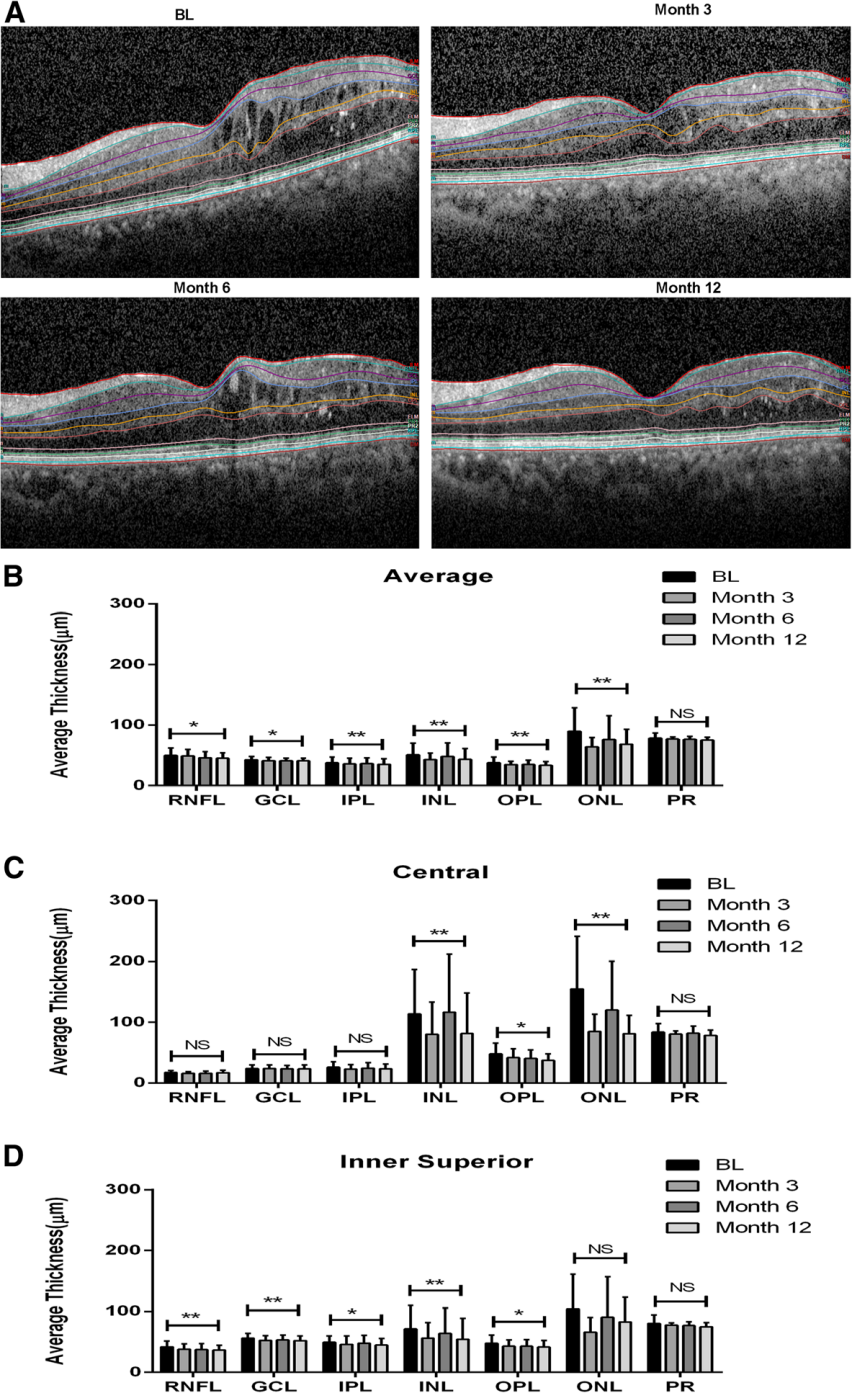
Individual layer thickness changes in study eye from baseline (BL) to 1 year of follow-up. **a** Representative optical coherence tomogram scans showing layer segmentation at baseline (BL) and at one-year (1-year) follow-up. Two-sided paired t-test was performed between baseline and month 12 (*p ≤ 0.05; **p ≤ 0.01; NS = not significant). Retinal layer thickness from BL to 12 months of continuous treatment in average changes (**b**), central subfield (**c**), and the inner superior region from inner ring (**d**) of the ETDRS grid. RNFL, retinal nerve fiber layer; GCL, ganglion cell layer; IPL, inner plexiform layer; INL, inner nuclear layer; OPL, outer plexiform layer; ONL, outer nuclear layer; PR, photoreceptor–RPE complex (Bruch membrane to external limiting membrane)

The treatment effect of conbercept is reflected in decrease in thickness in each individual retinal layer in 1 year of treatment. The average thickness of all layers became significantly thinner (*p* < 0.05). In the central subfield area, all the layer thicknesses were decreased while the INL, OPL and ONL layers had significant changes. Furthermore, when analyzing inner and outer ring, we found the inner superior section had the most significant changes in the most layers (Fig. 1). The layer thickness changes of the other regions are illustrated in Additional file 1: Figures S1 and S2.

### Correlation of individual layer changes with BCVA gain in different regions

**Fig. 2:**
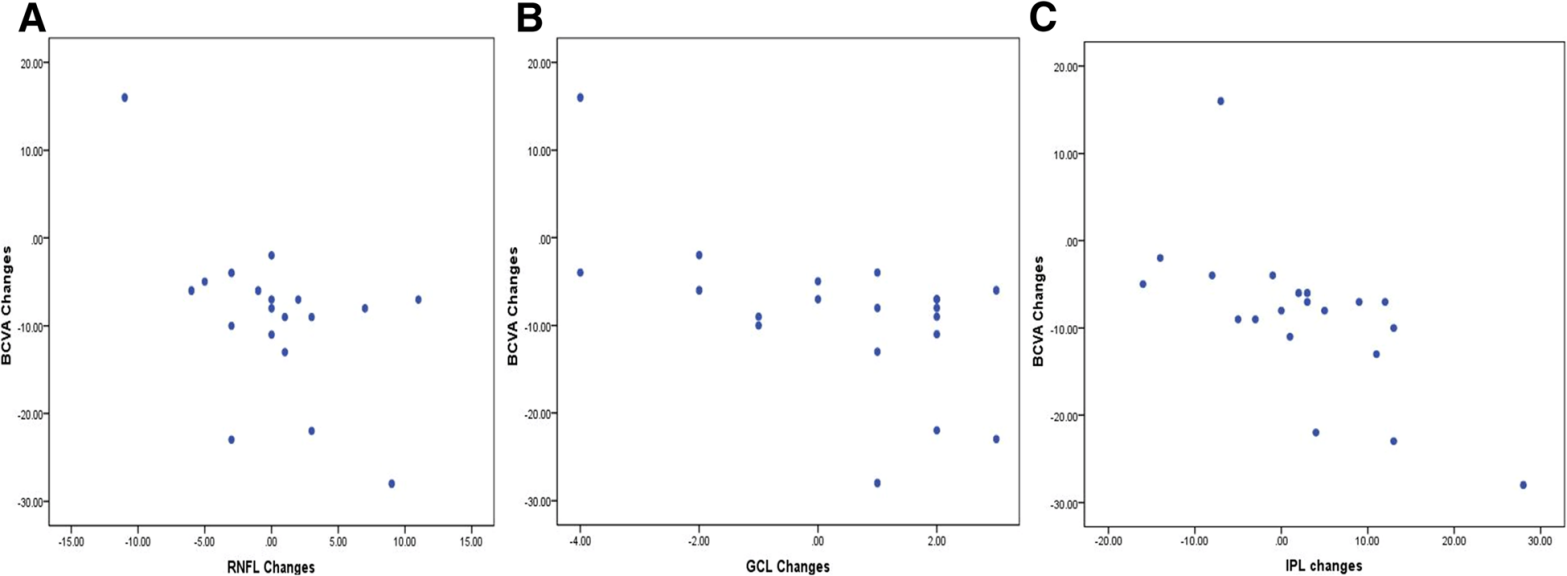
Correlation of individual layer thickness changes with BCVA gains after one-year treatment in central subfield. Scatterplots show the individual layer thickness changes in central subfield correlated with BCVA gains from baseline to one-year follow-up. RNFL, retinal nerve fiber layer (**a**); GCL, ganglion cell layer (**b**); IPL, inner plexiform layer (**c**)

The correlations of individual layer changes and other potentially important explanatory variables with BCVA gains were explored (Table 2). Decrease in GCL, IPL and RNFL thickness in central subfield corrected well with BCVA gain. (*r* = 0.591,0.663 and 0.558, respectively, Fig. 2). Besides, nasal part of IPL thickness both in inner ring and outer ring correlated well with BCVA gain (*r* = 0.552 and 0.678), while other layers did not show good correlation with BCVA gain in nasal part.

**Table 2:** Correlation for BCVA gain after 1 year conbercept treatment and individual layer thickness changes from different regions

Region	RNFL		GCL		IPL		INL		OPL		ONL		PR	
	Correlation Coefficient r	*p*	Correlation Coefficient r	*p*	Correlation Coefficient r	*p*	Correlation Coefficient r	*p*	Correlation Coefficient r	*p*	Correlation Coefficient r	*p*	Correlation Coefficient r	*p*
Average	0.389	0.090	0.415	0.069	0.473	**0.035**	0.411	0.072	0.371	0.107	0.495	**0.026**	0.190	0.422
Central	0.558	**0.011**	0.591	**0.006**	0.663	**0.001**	0.116	0.626	0.458	**0.042**	0.459	**0.019**	0.288	0.218
I.S.	0.478	**0.033**	0.039	0.869	0.017	0.943	0.322	0.167	0.35	0.131	0.429	0.059	0.115	0.628
I.I.	0.003	0.990	0.215	0.362	0.291	0.214	0.338	0.145	0.064	0.789	0.465	**0.039**	0.159	0.504
I.N.	0.402	0.079	0.01	0.967	0.552	**0.012**	0.206	0.384	0.113	0.635	0.494	**0.027**	0.212	0.370
I.T.	0.453	**0.045**	0.088	0.711	0.283	0.226	0.232	0.326	0.231	0.326	0.363	0.116	0.163	0.492
O.S.	0.44	0.052	0.563	**0.010**	0.137	0.563	0.080	0.736	0.441	0.052	0.378	0.100	0.469	**0.037**
O.I.	0.412	0.071	0.408	0.074	0.678	**0.001**	0.604	**0.005**	0.344	0.137	0.469	**0.037**	0.225	0.341
O.N.	0.234	0.322	0.227	0.336	0.527	**0.017**	0.488	**0.029**	0.300	0.198	0.444	**0.049**	0.183	0.441
O.T.	0.449	**0.047**	0.357	0.123	0.024	0.918	0.247	0.295	0.488	**0.029**	0.218	0.356	0.473	**0.035**

Significant *p* values are in bold

Region: Standard EDTRS grid are used with central subfield (*r* = 0.5 mm) (Central), inner ring(*r* = 0.5–1.5 mm) (I), outer ring(*r* = 1.5-3 mm) (O). Inner ring and outer ring are divided into four parts: superior part(S), inferior part (I), nasal part (N) and temporal part (T). I.S. stand for superior part of inner ring and so are the others

Dependent variable: Best-corrected vision acuity (BCVA) gain at the Final Visit

Explanatory variables: *RNFL* 1-year retinal nerve fiber layer thickness decrease, *GCL* 1-year ganglion cell layer thickness decrease, *IPL* 1-year inner plexiform layer thickness decrease, *INL* 1-year inner nuclear layer thickness decrease, *OPL* 1-year outer plexiform layer thickness decrease, *ONL* 1-year outer nuclear layer thickness decrease, *PR* 1-year photoreceptor-RPE-complex thickness decrease (external limiting membrane to Bruch’s membrane)

A multivariate analysis was further conducted for these subsets of data. In the univariate analysis, the decreases in some individual retinal layers’ thickness in central subfield were associated with larger BCVA gains. These findings were confirmed in the multiple linear regression (Table 3). In our cases, the best multivariate linear mode for predicting the BCVA gain included the thickness decreases of the IPL and GCL in the central subfield. The coefficient of determination R^2^ for this model was 0.544, outperforming all the other models. In this model, decrease in GCL and IPL were important factors that associated with final BCVA gain (*p* = 0.022 and 0.005, respectively).

**Table 3:** Multivariate linear regression analysis of factors with influence on BCVA gain after 1 year conbercept treatment in central subfield

	Univariate Analysis		Multivariate Analysis (Adjusted *R*^*2*^ = 0.544) ^a^		
	t	*p*	Unstandardized Regression Coefficient B	Standardized Regression Coefficient β	*p*
Constant			6.812		**< 0.001**
BL BCVA	0.509	0.617			
IVTs	0.695	0.496			
RNFL	−2.851	**0.011**			
GCL	−3.105	**0.006**	1.752	0.414	**0.022**
IPL	−3.756	**0.001**	0.455	0.523	**0.005**
INL	−0.495	0.626			
OPL	−2.184	**0.042**			
ONL	−2.585	**0.019**			
PR	−1.276	0.218			

Significant *p* values are in bold. t = β/ Standard Error (SE)

Dependent variable: Best-corrected vision acuity (BCVA) gain at the Final Visit

Explanatory variables: *BL BCVA* Baseline best-corrected visual acuity, *IVTs* Number of intravitreal injections, *RNFL* 1-year retinal nerve fiber layer thickness decrease, *GCL* 1-year ganglion cell layer thickness decrease, *IPL* 1-year inner plexiform layer thickness decrease, *INL* 1-year inner nuclear layer thickness decrease, *OPL* 1-year outer plexiform layer thickness decrease, *ONL* 1-year outer nuclear layer thickness decrease, *PR* 1-year photoreceptor-RPE-complex thickness decrease (external limiting membrane to Bruch’s membrane)

^a^Adjusted coefficient of multiple determination

### Correlation of microperimetry changes with BCVA gain in different regions

To further analyze the possible reason of this phenomenon, microperimetry analysis were performed after injection. The mean baseline visual acuity (EDTRS) of the treated eye increased by an average of 9.35 letters to 66.85 ± 10.17 letters at month 3(*p* < 0.001). Baseline mean macular sensitivity in different regions were 4.60 ± 2.95db (central subfield), together with 8.59 ± 3.92 db (superior), 8.79 ± 4.54 db (inferior), 9.80 ± 4.92 db (nasal) and 9.09 ± 4.19 db (temporal) in inner ring. Only increase of mean sensitivity in central subfield corrected well with BCVA gain (*r* = 0.531, *p* = 0.016) (Table 4).

**Table 4:** Correlation for BCVA gain after three conbercept treatment and mean sensitivity changes from different regions

Region	Baseline (db)	3 months(db)	Changes (db)	Correlation Coefficient r	*p*
Average	8.17 ± 3.66	9.51 ± 4.18	1.33 ± 2.00	0.433	0.056
Central	4.60 ± 2.95	5.87 ± 3.68	1.27 ± 2.35	0.531	**0.016**
I.S.	8.59 ± 3.92	9.86 ± 4.34	1.28 ± 1.93	0.014	0.954
I.I.	8.79 ± 4.54	10.56 ± 4.81	1.78 ± 2.66	0.409	0.073
I.N.	9.80 ± 4.92	11.15 ± 4.76	1.35 ± 2.71	0.444	0.05
I.T.	9.09 ± 4.19	10.09 ± 4.85	1.00 ± 2.27	0.338	0.144

Significant *p* values are in bold

Region: Standard EDTRS grid are used with central subfield (*r* = 0.5 mm) (Central), inner ring(*r* = 0.5–1.5 mm) (I), Inner ring and outer ring are divided into four parts: superior part (S), inferior part (I), nasal part (N) and temporal part (T). I.S. stand for superior part of inner ring and so are the others

Dependent variable: *BCVA* Best-corrected vision acuity gain at the 3 months Visit

Explanatory variables: changes of mean sensitivity in 3 months

## Discussion

Intravitreal conbercept has been shown to be effective in the treatment of proliferative diabetic retinopathy and age-related macular degeneration, resulting in BCVA improvement and central retinal thickness reduction in the treated eyes [14, 19]. The anti-VEGF drug conbercept was found to suppress the high glucose-induced migration and sprouting of human retinal endothelial cells by blocking VEGF and PIGF, thereby reconstructing capillary integrity [20]. Decreased vascular leakage lessens retinal fluid. Retinal layer thickness reduction would be the obvious result and is in fact detected in most layers and associated with visual acuity improvement. We found that the decreases in GCL and IPL were the best correlated with BCVA gain, although the retinal thickness decreased the most in the INL and ONL layers, where cystoid space and swelling are mainly located [21]. It could be explained by the neural recovery in the GCL and IPL layers. The RGC recovery was further proved in the Microperimetry results as the mean sensitivity gain in central subfield was correlated with BCVA gain after anti-VEGF injection.

Visual acuity gain was best correlated with the decreases in the GCL and IPL layer thickness. In our retrospective study, the changes in individual layer thickness from fellow eye were measured to compare the damage from the same level of hyperglycemia to the study eye, in which the center subfield and inner ring did not have significant changes (Additional file 1: Figure S3). Although GCL degeneration in diabetic patients has been previously described as an early-onset issue accompanying hyperglycemia [22] and microvascular abnormalities [23], our findings suggested that the effectiveness of conbercept in reducing edema which suggests possible intraretinal fluid accumulation [24]. Conbercept might provide benefit on neural recovery in the GCL and IPL layers by improving the metabolic supply with the resolution of subretinal fluid.

Interestingly, our study showed that the layer thickness of ONL, INL and OPL had significant changes during 1 year while these layers did not significant associate with the final BCVA gain. This might be explained as follows: in the early stages of DME development, a thickness increase occurs predominantly in the INL, probably due to colocalization of the deep retinal vascular net and the extracellular accumulation of fluid due to alterations of the blood-retinal barrier in these retinal capillaries [24]. The development of clinical macular edema in diabetes might be associated with structural damage of the remaining retinal layers, which allows an increased accumulation of fluid in the retinal layers located in ONL [25]. We suggested that there might be more primary nuclear damage in the baseline of our study. Nuclear cells that have already gone into apoptosis will not recover through resolution of subretinal fluid. Our hypothesis was further proved in the model used to predict final vision, where the decreases in the INL and ONL layers had negative correlations with final vision.

A ceiling effect was also found in the baseline BCVA, which contributes negatively in the model predicting visual acuity gain. This finding indicates that eyes with better baseline visual acuity gain fewer for there is less room for improvement [26]. Another study also suggested patients with better visual acuity at baseline gained fewer letters than patients with low visual acuity at baseline [27].

There are some limitations in this study. First, the retrospective nature and relatively small sample size might have hindered the potential associations. Furthermore, the pathologic conditions in retina cause the automated segmentation unreliable [28]. The analysis algorithms are manufacturer-specific, which differ between devices [29]. Manual correction is necessary to add a subjective component to the quantification. Better segmentation software will be needed for a large population study.

## Conclusion

To our knowledge, this is the first study to segment seven individual layers in the center retina during conbercept therapy, measuring treatment response both in BCVA and microperimetry respects. Edema reduction of GCL and IPL layer after conbercept therapy had the strongest correlation with recovery in visual acuity and sensitivity. Segmentation of ganglion cell layer and inner plexiform layer seemed to be of greater clinical importance for monitoring treatment response of conbercept therapy in DME patients than other layers.

## Additional file

Additional file 1: **Figure S1.** Individual layers thickness changes in study eye from baseline (BL) to 1 year follow-up. **Figure S2.** Individual layersthickness changes in study eye from baseline (BL) to 1 year follow-up. **Figure S3.** Individual layer thickness changes in fellow eye from baseline (BL) to 1-year follow-up. (DOCX 823 kb)

## Data Availability

The datasets used and/or analyzed during the current study are available from the corresponding author on reasonable request.

## Abbreviations

BCVA: Best-corrected visual acuity
DME: Diabetic macular edema
DR: Diabetic retinopathy
ETDRS: Early Treatment Diabetic Retinopathy Study
GCL: Ganglion cell layer
INL: Inner nuclear layer
IPL: Inner plexiform layer
ONL: Outer nuclear layer
ONL: Outer nuclear layer
PlGF: Placental growth factor
PR: Photoreceptor–retinal pigment epithelium complex
PRN: Pro re nata
RGC: Retinal ganglial cell
RNFL: Retinal nerve fiber layer
SD-OCT: Spectral domain optical coherence tomography
VEGF: Vascular endothelial growth factor
