# Supplementary material for: Correlation of retinal layer changes with vision gain in diabetic macular edema during conbercept treatment
Source: BMC Ophthalmol. 2019 May 31;19:123. doi: 10.1186/s12886-019-1131-0 (PMC6544971; doi:10.1186/s12886-019-1131-0)
Supplement: Supplementary file 1 — Figure S1. Individual layers thickness changes in study eye from baseline (BL) to 1 year follow-up. Figure S2. Individual layersthickness changes in study eye from baseline (BL) to 1 year follow-up. Figure S3. Individual layer thickness changes in fellow eye from baseline (BL) to 1-year follow-up. (DOCX 823 kb) [file 12886_2019_1131_MOESM1_ESM.docx]

Additional file 1





Figure S1 Individual layers thickness changes in study eye from baseline (BL) to one year follow-up. Two-sided paired t-test was performed between baseline and month 12 (*p≤0.05; **p≤0.01; NS=not significant). Retinal layer thickness from BL to 12 months of continuous treatment in the superior (A), inferior (B), nasal (C) and temporal (D) region from inner ring of the ETDRS grid. RNFL, retinal nerve fiber layer; GCL, ganglion cell layer; IPL, inner plexiform layer; INL, inner nuclear layer; OPL, outer plexiform layer; ONL, outer nuclear layer; PR, photoreceptor–RPE complex (Bruch membrane to external limiting membrane)





Figure S2 Individual layers thickness changes in study eye from baseline (BL) to one year follow-up. Two-sided paired t-test was performed between baseline and month 12 (*p≤0.05; **p≤0.01; NS=not significant). Retinal layer thickness from BL to 12 months of continuous treatment in the superior (A), inferior (B), nasal (C) and temporal (D) region from outer ring of the ETDRS grid. RNFL, retinal nerve fiber layer; GCL, ganglion cell layer; IPL, inner plexiform layer; INL, inner nuclear layer; OPL, outer plexiform layer; ONL, outer nuclear layer; PR, photoreceptor–RPE complex (Bruch membrane to external limiting membrane)


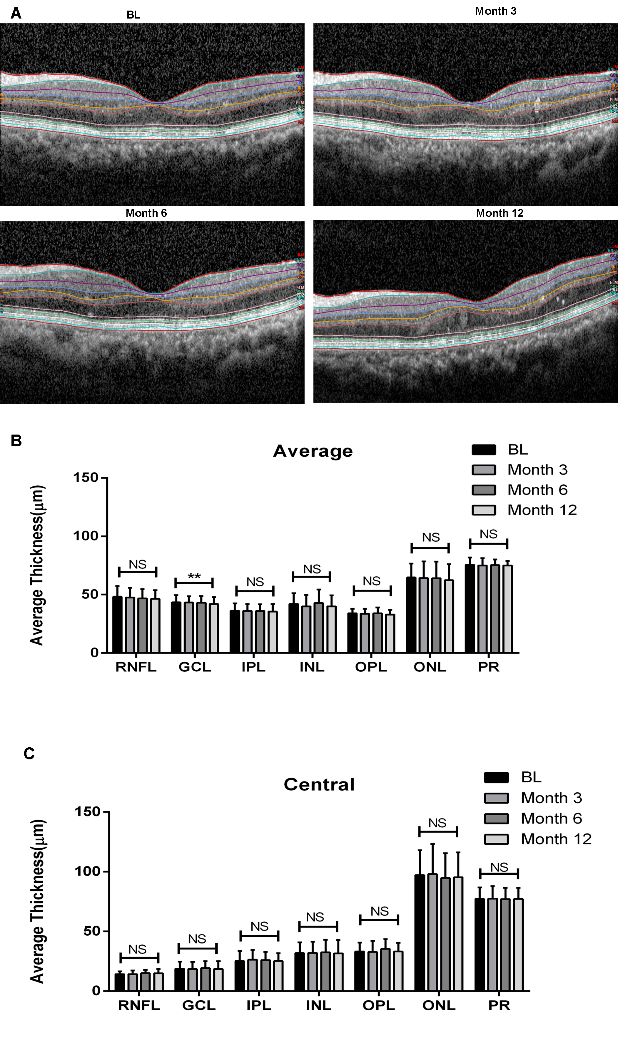


Figure S3. Individual layer thickness changes in fellow eye from baseline (BL) to one-year follow-up. (A) Representative optical coherence tomogram scans showing layer segmentation at baseline (BL) and at one-year (1-year) follow-up. Two-sided paired t-test was performed between baseline and month 12 (**p≤0.01; NS=not significant). Retinal layer thickness from BL to 12 months of continuous treatment in average changes (B), central subfield (C) of the ETDRS grid. RNFL, retinal nerve fiber layer; GCL, ganglion cell layer; IPL, inner plexiform layer; INL, inner nuclear layer; OPL, outer plexiform layer; ONL, outer nuclear layer; PR, photoreceptor–RPE complex (Bruch membrane to external limiting membrane).
